# Did the 1918 influenza cause the twentieth century cardiovascular mortality epidemic in the United States?

**DOI:** 10.7717/peerj.2531

**Published:** 2016-10-04

**Authors:** Steven Tate, Jamie J. Namkung, Andrew Noymer

**Affiliations:** 1 Pritzker School of Medicine, University of Chicago, Chicago, IL, United States; 2 Program in Public Health, University of California, Irvine, CA, United States; 3 Department of Population Health and Disease Prevention, University of California, Irvine, CA, United States

**Keywords:** Heart disease, Influenza, Epidemiology, Demography, Public health

## Abstract

During most of the twentieth century, cardiovascular mortality increased in the United States while other causes of death declined. By 1958, the age-standardized death rate (ASDR) for cardiovascular causes for females was 1.84 times that for all other causes, *combined* (and, for males, 1.79×). Although contemporary observers believed that cardiovascular mortality would remain high, the late 1950s and early 1960s turned out to be the peak of a roughly 70-year epidemic. By 1988 for females (1986 for males), a spectacular decline had occurred, wherein the ASDR for cardiovascular causes was less than that for other causes combined. We discuss this phenomenon from a demographic point of view. We also test a hypothesis from the literature, that the 1918 influenza pandemic caused the cardiovascular mortality epidemic; we fail to find support.

## Introduction

The twentieth century witnessed a slow-building increase of heart disease mortality, followed by a fall (Stallones, 1980); this pattern has been called an “epidemic” (Doll, 1987; Marmot, 1992). The decline of heart disease mortality in the last 50 years remains incompletely explained (Wilmot et al., 2015)—for instance, it occurred during an increase in obesity (Flegal, 2005; Carroll et al., 2015), which is thought to be a major risk factor for cardiovascular mortality (Flegal et al., 2007; Lavie, Milani & Ventura, 2009; Marinou et al., 2010). Azambuja & Duncan (2002a) proposed a creative hypothesis, that the 1918 influenza pandemic can explain this heart disease epidemic. We test the influenza hypothesis using historical data on heart disease mortality in the United States, disaggregated by age, sex, and period. Our analysis finds no support for the hypothesized role of the 1918 influenza pandemic. We begin by reviewing the history of American heart disease mortality, followed by an exploration of the influenza hypothesis in light of age-, sex- and period-specific mortality data. We then describe and implement a cohort test of the influenza hypothesis, using pre/post-1918 birth cohorts and data on heart disease mortality.

**Figure 1:**
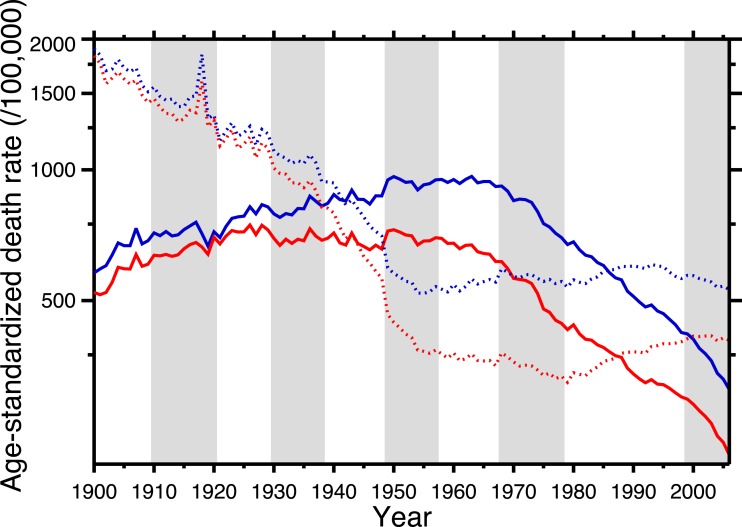
Age-standardized death rates, United States Death Registration Area (1900–32) and United States (1933–2006). Solid lines: heart disease. Dotted lines: all other causes excluding external causes (see text). Males, blue; females, red. Rates calculated by the authors from data listed in the Appendix. Alternating shading marks disease classification from ICD-1 to ICD-10. Year 2000 standard population used.

Figure 1 shows the age-standardized death rate (ASDR) for heart disease (solid lines), 1900–2006, for the United States, for males and females. This illustrates the long-term epidemic pattern of heart disease mortality in the United States in the twentieth century. Figure 1 also shows the age-standardized death rate for all other causes combined, excluding accidents, homicide, and suicide (dotted lines). Data sources are listed in Appendix, as is a tabulation of which ICD codes comprise heart disease (we define heart disease broadly, including the major cardiovascular diseases). This data set, which is available in the Supplemental Information, is potentially useful for others studying heart disease in the twentieth century.

The ASDR for non-cardio mortality shows a consistent decline, 1900–1955 (punctuated by the 1918 influenza pandemic), followed by a plateau and slight increase, 1965–2006. On the other hand, heart disease mortality shows a slow increase through the late 1940s, followed by a plateau until the late 1960s, and then a steep decline. Remarkably, from the early-1940s to the mid-1980s (i.e., where the solid lines are above the dotted lines in Fig. 1), the age-standardized death rate for cardiac causes exceeded that for all other causes combined (excluding violent causes). Indeed, Fig. 1 demonstrates that the decline in nonviolent all-cause mortality in the last 30 years is driven by the decline in cardiac mortality.

Azambuja & Duncan (2002a) proposed a bold and original hypothesis to explain the nearly century-long cardiovascular epidemic. The idea is elegant for its parsimony: exposure to the pandemic strain of the 1918 influenza virus preconditioned the survivors for later heart disease mortality. They postulate that the pandemic not only killed, in the United States, over half a million people (Johnson & Mueller, 2002), but also weakened the survivors:

We thus hypothesize that whatever immuneinflammatory mechanism caused a sex and age mortality pattern in 1918–1919 which differed from that of incidence of respiratory symptoms during the pandemic (see Fig. 1) also “primed” survivors in a similar fashion, predisposing them to future development of CHD. If that were the case, then the relative distribution of influenza-related deaths among individuals ages 15–49 in 1918–1919 (a proxy for the distribution of some particular kind of immune-inflammatory response to infection across the range of exposed birth cohorts) should predict the occurrence of CHD mortality in survivors from the corresponding birth cohorts (from about 1870 to 1915) in the subsequent years (Azambuja & Duncan, 2002a, pp. 558–559).

This neatly explains the rising side of the heart disease mortality epidemic. Cohort replacement takes care of the decline: “Since the flu epidemic was restricted (to 1918–19), new generations of unexposed individuals were then responsible for the later decline in CHD mortality” (Castilho & Gouveia, 2002; see also Sichieri, 2002; Ferreira, 2002; Werneck, 2002; Azambuja & Duncan, 2002b). Note, in Fig. 1, that the epidemic of cardiovascular mortality begins (although ambiguously) after 1919; this is congruent with the with the hypothesis. Azambuja & Levins (2007), and Azambuja (2008) discuss similar data. The pandemic hypothesis is based on aggregate data, and in the following section we similarly use aggregate data (albeit finer-grained) to test it.

## Results

**Figure 2:**
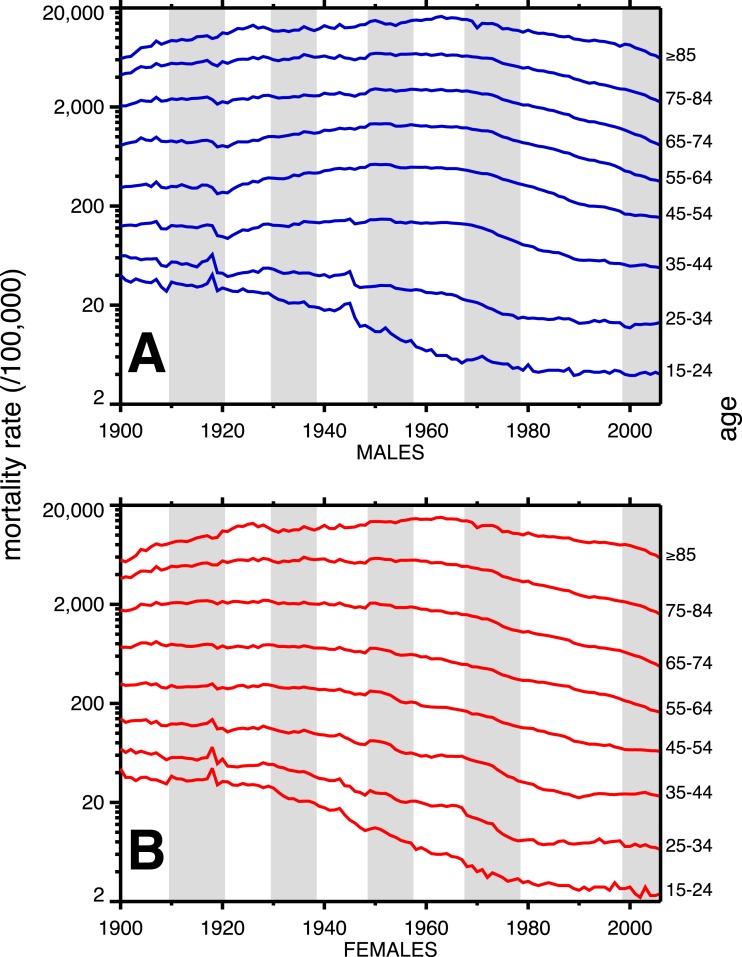
Heart disease age-specific death rates, United States Death Registration Area (1900–32) and United States (1933–2006). (A) Males, (B) females. Age groups are labeled on the right-hand vertical axes. Data sources and component causes listed in the Appendix. Alternating shading marks disease classification from ICD-1 to ICD-10.

**Figure 3:**
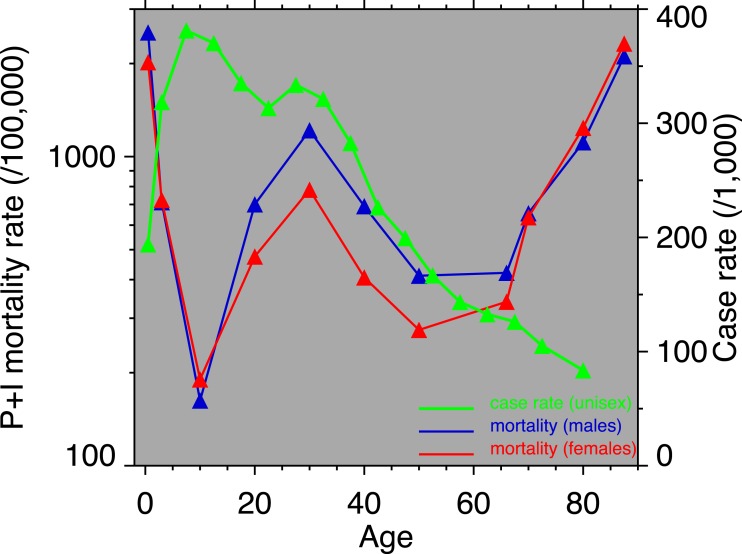
Pneumonia and influenza age-mortality profile, United States Death Registration Area, 1918. (Left vertical axis. Males, blue; females, red. From US Department of Health, Education, and Welfare (1956).) And influenza case rate or “attack rate.” (Right vertical axis, green. From Frost (1919).)

A breakdown, by age, of heart disease mortality in the same time span is presented in Fig. 2. The epidemic pattern depicted in Fig. 1 is present in ages 35 and above (more for males), but is more subdued when viewed as age-specific data slices. The year 1918 (presumably related to the influenza pandemic, since it was such a large and unusual event) is a pivot point in the heart disease data, especially for males, and most strongly in age groups 15–24 and 25–34. Not coincidentally, these were the peak ages of pandemic mortality (cf. Fig. 3).

Coincident with the pandemic, heart disease death rates go up, perhaps due to classification errors or comorbidities. Heart disease death rates then fall steeply in 1919. For example, for men aged 25–34 the cardiac death rate fell from the highest value in the twentieth century, in 1918, to the lowest value to date in the twentieth century, in 1919 (64.8 to 42.4 per 100,000). This has been characterized elsewhere as a harvesting effect, whereby those who died in the 1918 flu (whether their deaths were recorded as influenza or not) were sicker than average, therefore making a healthier than average surviving population in 1919, with lower death rates compared to the staus quo ante (Noymer, 2009).

In older age groups, especially 35–44, 45–54, and 55–64, and more for males, the post-1918 dip in cardiovascular death rates is followed by a rebound, which can be regarded as the beginning of the twentieth century heart disease epidemic. Note that the changes in heart disease mortality in the wake of the 1918 flu are age-dependent, and, to a large extent, sex dependent, with females showing no similar changes at ages 45 and above in 1919 and thereafter.

The alternating shaded and unshaded vertical bands in Figs. 1 and 2 denote changes in the ICD (International Classification of Diseases) version number, from 1 to 10 (Hetzel, 1997). No pattern changes appear to be due to succession of ICD versions, with the possible exception of the transition from ICD 5 to 6 in 1949, in which there is an uptick in mortality rates at ages 35–84 (somewhat more pronounced for women), in the year of the change. Another noteworthy pattern is the sharp decline, more for males, in cardiovascular mortality below age 35, around 1945. This is due to declines in rheumatic heart disease mortality associated with the invention of antibiotics (Crimmins, 1981).

Figure 3 shows the age-profiles of 1918 influenza mortality and of incidence (i.e., morbidity). The age groups for which heart disease rises the most, post-1920, are 35 and older (Fig. 2), which is above the age of peak impact of the 1918 influenza as shown in Fig. 3. This already casts some doubt on the 1918 flu/cardiovascular hypothesis. Consider closely males (i.e., Fig. 2A) at ages 15–24, 25–34, 35–44 and 45–54. All these ages should respond similarly, if the 1918 influenza were a watershed moment in cardiovascular mortality. It is important to note that in Fig. 3, *survivors* matter more than the dead (cf. Sheps, 1958), so the key curve is the incidence.^1^ 1Technically, it is the incidence net of survivorship, but given the differing scales involved (compare the left and right axis of Fig. 3), incidence is a good first-order approximation.What is especially inconsistent with the influenza hypothesis is that cardiovascular mortality declines in the wake of the flu for ages 15–24 and 25–34, whereas it increases for 35–44 and 45–54. These cohorts all experienced unusual flu mortality in 1918, which is the marker, according to the influenza hypothesis, for an enhanced heart disease response. The heterogeneous response is not expected under the hypothesis. What is more, women, who were affected by the flu, albeit somewhat less than men, show no after-effects of cardiovascular mortality in the wake of the 1918 pandemic (Fig. 2B).

While the patterns in Fig. 2 are complex, we posit that the twentieth century history of cardiovascular mortality is dominated by period effects—not by the cohort effects predicted by the influenza hypothesis. For example, the rise and subsequent fall in heart disease mortality beginning in the 1920s happens similarly (indeed, almost parallel) across age groups. Cohort effects characteristically occur with a delay caused by aging. A cohort effect among those aged 20–24 in 1918 will be seen among those aged 30–34 in 1928 and those 40–44 in 1938, and so on. On the other hand, parallel lines (i.e., in Fig. 2) are suggestive of period effects, with some factor(s) influencing several age groups in the same way at the same time.

To assess how much the lines in Fig. 2 are moving in parallel, we performed Goodman–Grunfeld (G–G) tests for co-movement of time series with correction for serial correlation (Goodman & Grunfeld, 1961). Co-movement in this statistical test refers to correlations of signs of first differences of two series. That is to say, when one series goes up (year-to-year) that the other one does as well, and the same with declines. It is unusual for two mortality time series to be in perfect lock-step; this test for co-movement gives a statistical measure of the tendency for two series to co-move. Pure period effects (e.g., this year has lenient mortality, next year, severe, and so on) predict statistically-significant co-movements between the time series in Fig. 2.

Table 1 gives every pairwise Goodman–Grunfeld test of the time series in Fig. 2 (within sex). Reading across each row of Table 1 gives the G–G test statistic, comparing that mortality time series to older age groups. Reading down each column compares with younger age groups. Adjacent age groups are on the diagonal, which is always the strongest relationship, with the exception of females, age ≥ 85 (where the relationship is trivially stronger for co-movement with the 65–74 age group). Table 1 is highly suggestive that adjacent age groups are moving together, as period effects shape the time series—-indeed, most cells are significant (≥1.96), so, basically, the time series at all ages are moving *en masse* from year-to-year.

**Table 1:** Goodman–Grunfeld test statistics for co-movement of data series in Fig. 2. The test statistics follow a normal distribution, so any number ≥1.96 can be regarded as significant at the 5% level in a two-sided test.

	25–34	35–44	45–54	55–64	65–74	75–84	≥85
Males							
15–24	1.97	1.16	1.32	0.71	1.14	0.02	0.14
25–34		4.09	2.63	2.06	1.25	0.92	0.75
35–44			5.04	4.15	4.18	3.85	2.93
45–54				6.76	5.17	4.85	4.33
55–64					7.10	5.93	4.95
65–74						8.00	5.76
75–84							6.91
Females							
15–24	2.23	2.26	1.79	1.94	1.62	0.85	0.04
25–34		4.79	3.67	1.53	2.11	1.77	0.27
35–44			5.94	4.82	4.38	4.04	2.79
45–54				5.36	5.94	6.06	3.31
55–64					6.15	6.74	4.86
65–74						7.73	6.08
75–84							5.96

Table 1 supports period effects, but, except perhaps by implication, it does not rule out cohort effects. To explore cohort effects, we analyzed the co-movements of adjacent age groups, with lags. The diagonals of Table 1 coincide with this analysis with zero lag. In the uppermost left corner, males 15–24 co-move with males 25–34, with a G-G test statistic of 1.97. A lag of one year is a comparison of co-movement of the 15–24 age group with the 25–34 age group, but translated one year in time (with the younger age group moving up). For a lag of two, it’s a two year temporal shift, etc. For a lag of 10 years, we are comparing a group of individuals with themselves (modulo cohort survivorship). That is to say, with a 10-year lag, the time steps are 1920 to 1921 (then 1921 to 1922...) for the 15–24 age group, but, because of the lag, 1930 to 1931 (then 1931 to 1932...) for the 25–34 age group, and so on. Since those 15–24 in 1920 are 25–34 in 1930, the comparison with 10-year lag is, substantially, comparing the same group of people. Of all the lags we tested (1–20 years) cohort overlap peaks at 10 years, and is zero beyond 20 years. At lags other than 10 years, we are looking at mixtures of adjacent (10-year) birth cohorts. If there are are cohort effects, we expect to see a bar graph of the test statistics having a local or global maximum at a lag of 10.

**Figure 4:**
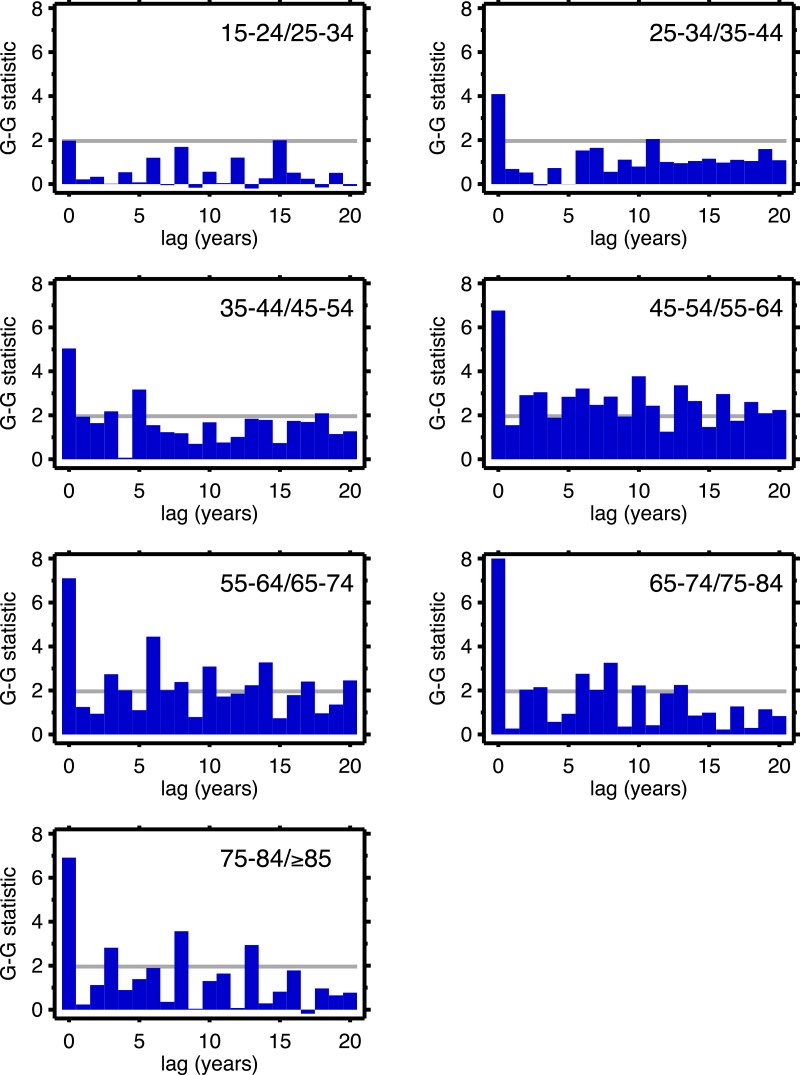
Goodman–Grunfeld test statistics for co-movement of *adjacent* age groups, under various lags. Males. Horizontal line represents conventional level of statistical significance. See text for ‘Discussion.’

**Figure 5:**
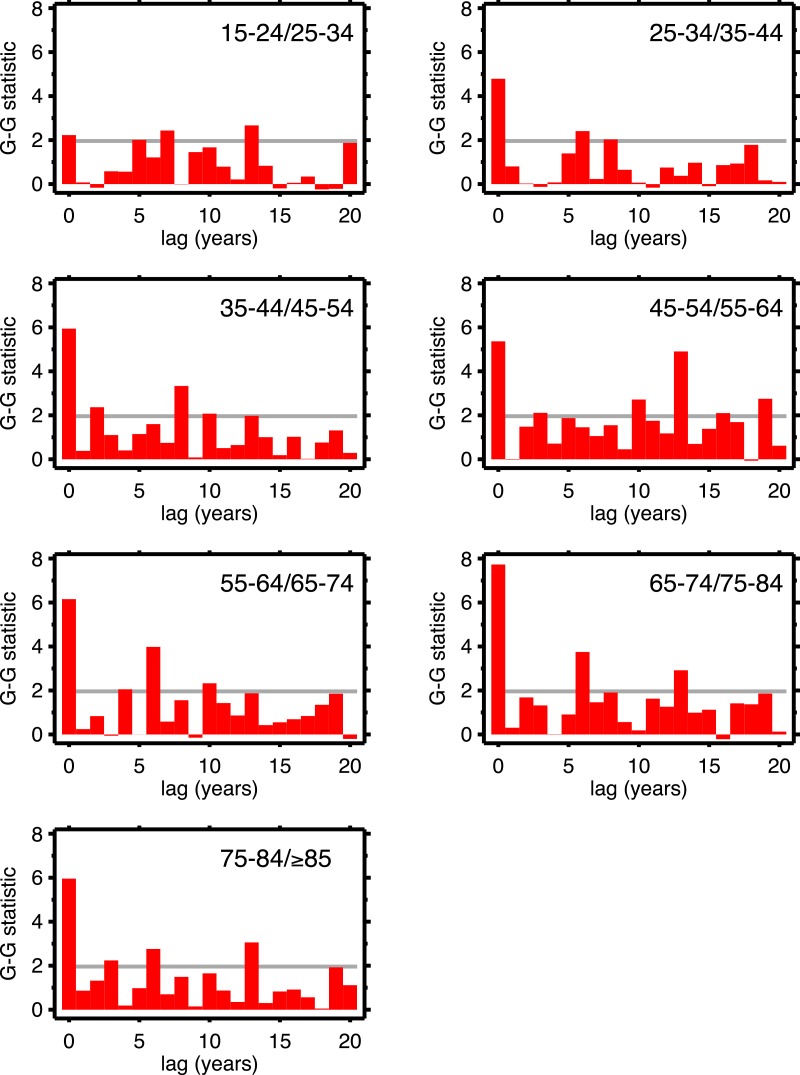
Goodman–Grunfeld test statistics for co-movement of *adjacent* age groups, under various lags. Females. Horizontal line represents conventional level of statistical significance. See text for ‘Discussion.’

Figures 4 (males) and 5 (females) are bar graphs of the G–G test statistic of age-adjacent cardiovascular mortality rates, at lags 0–20, as discussed above. For every age combination (separately by sex) across Figs. 4 and 5, the maximum co-movement occurs at lag 0, indicating that period effects predominate. What is more, the lags don’t show any preference for 10 years. The data in Fig. 2 are more consistent with period effects than with the cohort (viz influenza) hypothesis. These data are the same as have been used to argue in favor of the influenza hypothesis (e.g., Fig. 1 in Azambuja & Levins, 2007), except that they have been disaggregated by age.

We are alternately considering period, and cohort, perspectives, all the while stratifying by age and sex. This is not a unified age-period-cohort (A-P-C) framework in the formal way that term is used in demographic research (Hobcraft, Menken & Preston, 1982)). This is intentional, as the A-P-C approach has received justified criticism (e.g., Rodgers, 1982; Mason, 1991; Smith, 2004; Luo, 2013). The exploratory approach (Tukey, 1977) used herein, combined with the above G-G tests, seems more suited to the data and questions at hand.

## Discussion

The idea that diseases or exposures can affect individuals after a long delay, and can influence outcomes of seemingly-unrelated diseases, has a long pedigree (Derrick, 1927; Kermack, McKendrick & McKinlay, 1934; Frost, 1939; Merrell, 1947; Kuh & Davey Smith, 1993) discuss the history. The work of Barker and collaborators, on *in utero* exposures, is a special case of the larger phenomenon. This strand of work began in the 1980s (e.g., Barker & Osmond, 1986; Barker et al., 1989; Barker, 1990), and has spawned a huge literature of its own. The 1918 influenza has been fertile subject matter in this vein (Almond & Mazumder, 2005; Almond, 2006 (see also Brown & Thomas, 2013); Lin & Liu, 2014). Azambuja & Duncan’s (2002a) hypothesis that exposure to the 1918–19 influenza pandemic preconditioned later heart disease mortality also belongs in the category of early or delayed influences.

We analyzed data on cardiovascular death rates, by age and sex. Looking at all age groups and long time spans permits age, period, and cohort perspectives. We find salient evidence for period effects, with adjacent time series (e.g., 25–34 and 35–44) strongly co-moving, statistically. In other words, changes seen in one age group from year to year are almost always seen in the adjacent age group (and, indeed, in age groups beyond that).

The presence of period effects does not imply absence of cohort effects, since the two forces are not mutually exclusive. Suppose cohorts exposed to the 1918 influenza are somehow scarred such that they experience higher cardiovascular death rates. The upward trend of cardio death rates in the 1920s of 35–44 year-olds should be matched by a similar trend among 45–54 year-olds in the 1930s, and for 55–64 year-olds in the 1940s, and so on, at least until out-selection of the dead has diminished the putative scarring effect. By looking at 10-year age groups with 10-year lags, we looked at the mortality experiences of the same cohort over time. Under the null hypothesis of cohort effects, other lags should produce less strong co-movement, since the cohort overlap is much less (for example, 10-year age data and a 5-year lag). We see no preference for 10 years among all the lags in the G–G test statistics, which we interpret as lack of support for cohort effects.

This study has a number of limitations. Preston (1976) makes the point that cardiovascular diseases are especially subject to misclassificiation as causes of death. This invites the alternate hypothesis that the decline in cardiovascular mortality could be due to improvements in death classification which move similar deaths to non-cardiac causes. However, our broad definition of cardiovascular mortality (cf. Appendix) would seem to buffer against this. So does the fact, also noted by Preston (1976), that cardiovascular causes are often the replacement for causes such as “senility” that nowadays are regarded as so-called garbage codes (in other words, technical improvements over time can increase as well as diminish cardiovascular death counts).

The available data are in ten-year age groups, which are wider than ideal. Five-year age groups (unavailable in the historical data) would be preferable because they are more detailed than the data at our disposal, while still being wide enough to smooth digit-preferences (e.g., heaping of deaths of 75 year-olds in 1975) that plague single-year data. Individual-level data, such as a modern epidemiological cohort study, with measured exposure to the 1918 influenza, would be the best, but are unavailable. The influenza hypothesis as regards cardiovascular mortality was framed using aggregate data, and, we believe, can be tested further using aggregate data that is more granular (age groups). The 20th century cardiovascular mortality epidemic remains somewhat mysterious. The role of the rise and fall of tobacco smoking also should be considered; the timing and sex-specific aspects align with the patterns of cardiovascular mortality (Bongaarts, 2014).

## Conclusion

We find the hypothesis that the 1918 influenza pandemic played a key role in the 20th century cardiovascular mortality epidemic to be creative and original. However, it is not congruent with the available data on long-term changes in heart disease mortality, when arrayed by sex × age × period. A complete explanation of the twentieth century cardiovascular mortality epidemic in the United States remains elusive.

##  Supplemental Information

10.7717/peerj.2531/supp-1 Supplemental Information 1 All data used in the study

## Appendix: ICD codes for “heart disease”

See the table below:

**Table:**

Years	ICD	Codes used	Data source
1900–1909	1	47, 64–66, 77–86, 142	A
1910–1920	2	47, 64–66, 77–85, 142	A
1921–1929	3	51, 74, 75, 83, 87–90, 91b,c, 92–96, 151	A
1930–1938	4	56, 82, 90–95, 97–103	A
1939–1948	5	58, 83, 90–103	A
1949–1957	6	330–334, 400–468	A (1949–53); B (1954–57)
1958–1967	7	330–334, 400–468	B (1958–59); C (1960–67)
1968–1978	8	390–448	D
1979–1998	9	390–448	E
1999–2006	10	I00–I78	F
Data sources:			
A	US Department of Health, Education, and Welfare (1956)		
B	National Center for Health Statistics (2005)		
C	National Center for Health Statistics (2004a)		
D	National Center for Health Statistics (2004b)		
E	National Center for Health Statistics (2000)		
F	National Center for Health Statistics (2009)		

## Additional Information and Declarations

### Data Availability

The following information was supplied regarding data availability: The raw data has been supplied as Supplemental File.
